# A facial depression recognition method based on hybrid multi-head cross attention network

**DOI:** 10.3389/fnins.2023.1188434

**Published:** 2023-05-24

**Authors:** Yutong Li, Zhenyu Liu, Li Zhou, Xiaoyan Yuan, Zixuan Shangguan, Xiping Hu, Bin Hu

**Affiliations:** Gansu Provincial Key Laboratory of Wearable Computing, Lanzhou University, Lanzhou, China

**Keywords:** facial depression recognition, convolutional neural networks, attention mechanism, automatic depression estimation, end-to-end network

## Abstract

**Introduction:**

Deep-learn methods based on convolutional neural networks (CNNs) have demonstrated impressive performance in depression analysis. Nevertheless, some critical challenges need to be resolved in these methods: (1) It is still difficult for CNNs to learn long-range inductive biases in the low-level feature extraction of different facial regions because of the spatial locality. (2) It is difficult for a model with only a single attention head to concentrate on various parts of the face simultaneously, leading to less sensitivity to other important facial regions associated with depression. In the case of facial depression recognition, many of the clues come from a few areas of the face simultaneously, e.g., the mouth and eyes.

**Methods:**

To address these issues, we present an end-to-end integrated framework called Hybrid Multi-head Cross Attention Network (HMHN), which includes two stages. The first stage consists of the Grid-Wise Attention block (GWA) and Deep Feature Fusion block (DFF) for the low-level visual depression feature learning. In the second stage, we obtain the global representation by encoding high-order interactions among local features with Multi-head Cross Attention block (MAB) and Attention Fusion block (AFB).

**Results:**

We experimented on AVEC2013 and AVEC2014 depression datasets. The results of AVEC 2013 (RMSE = 7.38, MAE = 6.05) and AVEC 2014 (RMSE = 7.60, MAE = 6.01) demonstrated the efficacy of our method and outperformed most of the state-of-the-art video-based depression recognition approaches.

**Discussion:**

We proposed a deep learning hybrid model for depression recognition by capturing the higher-order interactions between the depression features of multiple facial regions, which can effectively reduce the error in depression recognition and gives great potential for clinical experiments.

## 1. Introduction

Major depressive disorder (MDD), also called depression, is one of the most common mental and mood disorders. It presents itself through depressed mood, pessimism, loss of attention and memory, self-denial, poor appetite, and decreased activity, among other symptoms. In addition, it can severely impact a person's thoughts, behaviors, work-life, and eating habits (Belmaker and Agam, 2008). With the increasing pressure of life, many people are suffering from depression. The World Health Organization (WHO) released data in 2007 stating that 350 million people worldwide suffered from depression. Moreover, in 2030, depression may overtake cardiovascular disease as the number one cause of disability, which means that depression has become a severe social health problem (World Health Organization, 2017). Unfortunately, there are no impactful clinical patterns for the diagnosis of depression due to personal and social development and other factors, which makes the diagnosis of depression complicated and subjective (Maj et al., 2020). Meanwhile, there are few professional psychiatrists in some developing countries, and the insufficient ratio of doctors to patients has become a major problem faced by mental health workers as well. Therefore, it is necessary to find objective parameter indicators to assist doctors in improving the current medical situation.

Studies have shown that depression alters various non-verbal behaviors (Ellgring, 2007), including psychomotor delays, insensitivity to emotional stimuli, and diminished positive and negative emotional responses, all of which can transfer information about depression levels (Cohn et al., 2009; Michalak et al., 2009; Canales et al., 2017). Especially, the face presents most of the people's non-verbal information, which leads to that as a characteristic indicator with high information content in the diagnosis of depression. Clinically, patients with depression often have reduced facial expression richness, drooping eyes, frowning, drooping mouth corners, reduced smile, and easy crying (Pampouchidou et al., 2020). Therefore, various researchers from the affective computing field have attempted to use facial changes as a biomarker to analyze the individual depression level and measured by the Beck Depression Inventory-II (BDI-II) score (McPherson and Martin, 2010), as presented in Table 1.

**Table 1:** The relation between the BDI-II cut-off scores and the depression severity level.

**BDI-II score**	**Severity level**
0–13	None or minimal
14–19	Mild
20–28	Moderate
29–63	Severe

Estimating the level of depression from facial images usually includes the following steps: (1) feature extraction and (2) regression (or classification). Among them, the task of feature extraction involves designing an effective depression representation that plays a significant role in facial depression recognition. At present, there are two main methods of feature extraction as follows: hand-crafted (Valstar et al., 2013, 2014; Wen et al., 2015) and deep-learned (Jan et al., 2017; Zhu et al., 2017; Al Jazaery and Guo, 2018; Zhou et al., 2020; Guo et al., 2021). For hand-crafted features, Local Phase Quantization (LPQ) and Local Gabor Binary Patterns from Three Orthogonal Planes (LGBP-TOP) are adopted as visual features for predicting the scale of depression (Valstar et al., 2013, 2014). However, these features are difficult to obtain accurate and subtle facial information (Song et al., 2018). Meanwhile, hand-crafted methods often involve a complex set of image processing steps, leading to relying heavily on expert knowledge (Ojala et al., 2002; Laptev et al., 2008; Meng and Pears, 2009). On the contrary, deep learning features do not rely on expert knowledge and complex manual design, which can capture and reveal high-level semantic features of faces. Zhou et al. (2020) propose a deep regression network to learn a depressive feature representation visually interpretably, and the result shows that the area near the eyes plays a crucial role in recognizing depression. Al Jazaery and Guo (2018) have automatically learned spatiotemporal features of facial regions at two different scales by using three-dimensional convolutional neural network (3D-CNN) and recurrent neural network (RNN), which can model the local and global spatiotemporal information from continuous facial expressions to predict depression levels.

**Figure 1:**
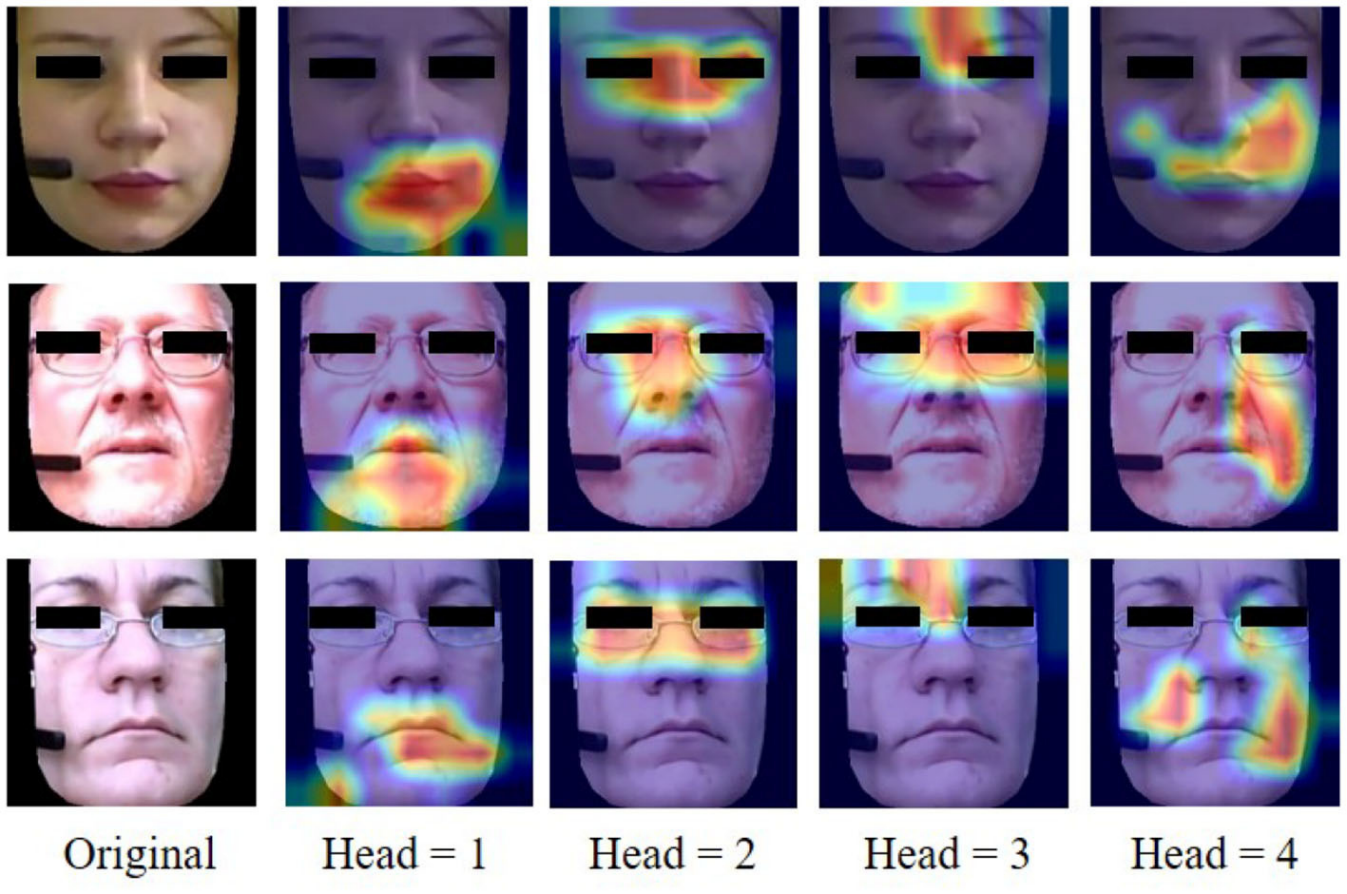
Example cases of visualization facial images with different cross-attention head. The first column is original facial images (BDI-II scores of 3, 16, and 44 from top to bottom), and the rest of the columns are generated by four cross-attention heads from HMHN.

However, most of the above methods do not further explore the local details. One unique aspect of facial depression recognition lies in the delicate contention between capturing the subtle local variations and obtaining a unified, holistic representation. Some recent studies focus on attention mechanisms to balance the local details and unified, holistic representation. For instance, He et al. (2021a) propose an integrated architecture called Deep Local-Global Attention Convolutional Neural Network (DLGA-CNN), which utilizes Convolutional Neural Network (CNN) with attention mechanism and weighted spatial pyramid pooling (WSPP) to model a local-global facial feature. Liu et al. (2023) design a global region-based network with part-and-relation attention, which learns the relation between part and global features. Niu et al. (2022) introduce an architecture using CNN and attention mechanism for automatic depression recognition by facial changes, and the performance surpasses most facial depression recognition methods. These methods focusing on attention mechanisms have achieved promising results by paying attention to facial details. Nevertheless, as shown in Figure 1, it is difficult for a model with only a single attention head to concentrate on various parts of the face simultaneously and just concentrate on one coarser image region, missing other important facial locations. Existing research results show that the differences in facial changes between depressed patients and healthy people are simultaneously manifested in multiple parts of the face (Schwartz et al., 1976; Scherer et al., 2013), such as eyebrows, eyes, cheeks, and mouth. Therefore, to mitigate the problems mentioned above, we propose a Hybrid Multi-Head Cross-Attention Network (HMHN), which implements multiple attention mechanisms to capture the high-order interactions between the local features of multiple facial regions by instantiating multiple attention heads.

**Figure 2:**
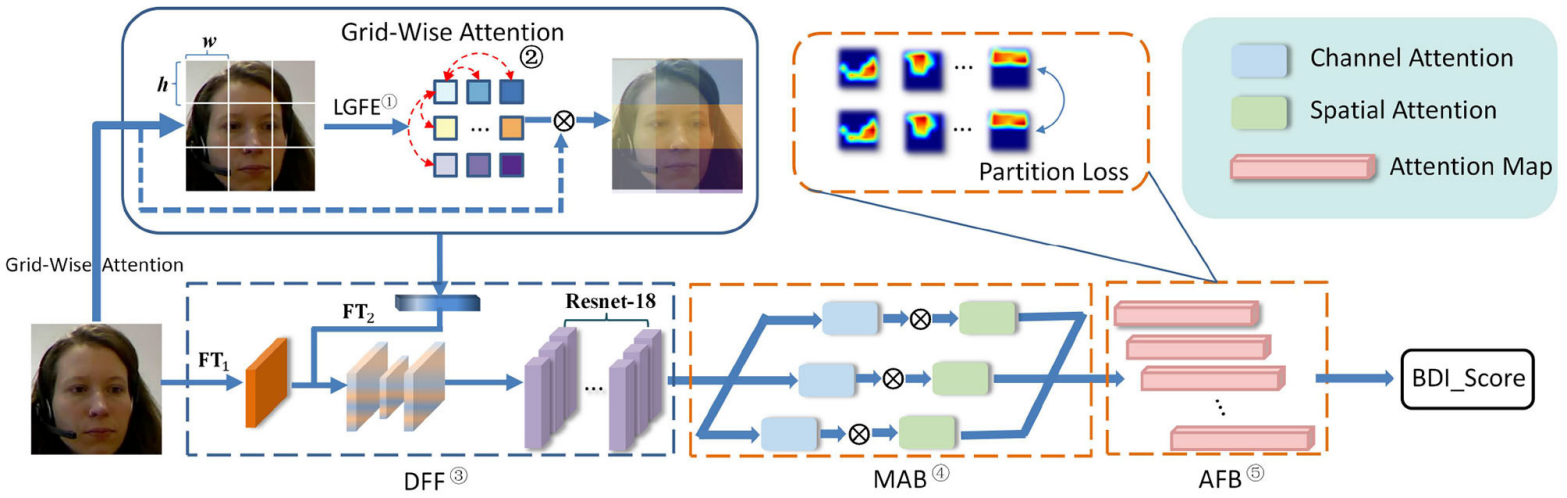
The framework of HMHN. Notations: ① Local Grid Feature Extraction, ② Grid-Wise Attention Calculation, ③ Deep Feature Fusion Block, ④ Multi-head cross Attention Block, and ⑤ Attention Fusion Block. DFF, deep feature fusion block; FT_*i*_, feature transformation network in DFF; MAB, multi-head cross attention block; AFB, attention fusion block.

More specifically, as shown in Figure 2, the HMHN consists of four components as follows: (1) Grid-Wise Attention Module (GWA), (2) Deep Feature Fusion Block (DFF), (3) Multi-head cross Attention Block (MAB), and (4) Attention Fusion Block (AFB). Concretely, GWA and DFF are designed to model the long-range dependencies among different regions of the low-level facial image. Next, MAB further measures the high-level detail features from multiple facial regions by combining multiple attention heads, consisting of spatial and channel attention. At the same time, the AFB module makes the attention maps extracted by the MAB focus on different regions, which enables the HMHN to capture several depression-related face regions simultaneously. Finally, AFB outputs the depression severity (BDI-II Score).

The main contributions of this study can be summarized as follows:

• We propose an integrated end-to-end framework HMHN, which effectively captures the facial dynamics information from multi-region as a non-verbal behavior measure for estimating the severity of depression scale.
• To regularize the convolutional parameter learning in the low-level feature extraction for facial depression recognition, we design grid-wise attention and DFF block, which can model long-range dependencies between different facial regions.
• To address the problem that a single attention module cannot adequately capture the subtle depression features of faces, we propose MAB and AFB. On the one hand, MAB further extracts high-level detail features. On the other hand, AFB is designed to capture multiple non-overlapping attention regions and fuse them to encode high-order interactions among local features.
• We conduct the compared experiments on two publicly benchmark depression datasets [i.e., AVEC 2013 (Valstar et al., 2013) and AVEC 2014 (Valstar et al., 2014) depression datasets]. The results demonstrate that our method is promising against several state-of-the-art alternative methods. Moreover, we also do an ablation study that specifically demonstrates the effectiveness of each component in our model.

The structure of the remaining chapters is provided as follows. We, first, briefly discussed the related work in Section 2, and the proposed depression recognition method is described in Section 3. Section 4 demonstrates the dataset and experimental settings. The results and discussions are presented in Section 5, and Section 6 concludes the study.

## 2. Related work

### 2.1. Hand-engineered methods

In the third and fourth Audio-Visual Emotion recognition Challenge depression sub-challenges (AVEC 2013/14), the datasets for depression level prediction are publicly released, which contributed notably to research on automatic depression detection. In the AVEC 2013 depression sub-challenges, they use the Local Phase Quantization (LPQ; Ojansivu and Heikkilä, 2008) feature descriptor as visual features to predict the BDI-II score. Cummins et al. (2013) investigate Space-Time Interest Points (STIP; Laptev et al., 2008) and Pyramid of Histogram of Gradient (PHOG; Bosch et al., 2007) descriptors for extraction of behavioral cues for depression analysis. Meng et al. (2013) propose to use Motion History Histogram (MHH) feature (Meng and Pears, 2009) to model motion in videos by improving the Motion History Image (MHI) in the field of action recognition, and the Partial Least Squares (PLS; De Jong, 1993) is employed for regression learning. Wen et al. (2015) propose to encode temporal information based on Local Phase Quantization from Three Orthogonal Plane (LPQ-TOP) features from sub-volumes of the facial region through discriminative mapping and decision fusion, and the recognition performance is further improved. The following research on the AVEC 2013 dataset relies on Median Robust Local Binary Patterns from Three Orthogonal Planes (MRLBP-TOP; He et al., 2018) and Local Second-Order Gradient Cross Pattern (LSOGCP; Niu et al., 2019). In the AVEC 2014 depression sub-challenges, the author extracted the Local Gabor Binary Pattern (LGBP; Zhang et al., 2005) feature from the XY-T place of video to predict the BDI-II score. In the study by Dhall and Goecke (2015), Local Binary Patterns (LBP) from three orthogonal plane (TOP) feature descriptors have been considered effective for predicting the scale of depression. In the study by Pérez Espinosa et al. (2014), they use dynamic facial features extracted by LGBP from Three Orthogonal Planes (LGBP-TOP) to predict depression level, another variant of LBP-TOP.

The above methods based on hand-crafted feature descriptors have some positive results in the field of depression recognition. However, they still have some limitations. For instance, hand-crafted features are highly dependent on expert knowledge and cannot extract complex semantic information.

### 2.2. Deep learning methods

As deep networks can extract deeper and more spatial inductive biases information, deep learning methods have gained their prevalence in facial depression recognition tasks. According to combined facial appearance with dynamic features (optical flow) in fully connected layers, Zhu et al. (2017) fine-tune to adopt deep models (GoogleNet), pre-trained on the CASIA (Yi et al., 2014) large facial dataset for predicting BDI scores from video data, and achieve positive performance on AVEC 2013 and AVEC 2014 depression datasets. Zhou et al. (2020) propose a multi-region DepressNet neural network by blending different facial regions on the basis of ResNet-50 (He et al., 2016), proving that the combination of multiple sub-models can improve the performance of depression recognition. In the study by De Melo et al. (2019), Melo et al. adopt a 2D-CNN and distribution learning to predict the BDI-II score from facial images. Similarly, many of the following works using pre-trained CNNs fine-tune their deep architectures on the AVEC 2013 and AVEC 2014 datasets to estimate and prediction (e.g., Kang et al., 2017; De Melo et al., 2020; He et al., 2022a). He et al. (2021a) combine the attention mechanism with CNN to construct an end-to-end depression recognition model named LGA-CNN. He et al. (2022b) also designed an end-to-end framework called the SAN to re-label the uncertain labels for automatic depression estimation. Niu et al. (2022) utilize a pre-trained ResNet-50 model to process video clips. They employed a graph convolution embedding block and a multi-scale vectorization block to capture and represent facial dynamics for predicting BDI-II scores, which reflect the severity of depression. Liu et al. (2023) propose an end-to-end depression recognition model called PRA-Net. They divide the input facial images into parts and calculate the feature weight of each part. Then, they combine the parts using a relation attention module. PRA-Net utilizes part-based and relation-based attention mechanisms to improve the model's performance.

To extract depression cues from the perspective of spatial structure and temporal changes, various studies have been proposed to model spatio-temporal information for depression recognition. Al Jazaery and Guo (2018) have automatically learned spatio-temporal features of face regions at two different scales by using 3D Convolutional Neural Network (C3D) and Recurrent Reural Network (RNN), which can model the local and global spatio-temporal information from continuous facial expressions to predict depression levels. De Melo et al. (2020) designed a novel 3D framework to learn spatio-temporal patterns by combining the full-face and local regions. Uddin et al. (2020) introduce a new two-stream network to model the sequence information from video data. In addition, the 3D-CNN is also used in the study by De Melo et al. (2021) and He et al. (2021b) to capture informative representations for analyzing the severity of depression. In contrast to the above methods, our HMHN achieves comparable results using only facial visual information.

As mentioned above, the existing approaches extract high-level representations of depression cues through CNN, but there are still some problems. First, most of these depression estimation methods are not end-to-end schemes, which increases the difficulty of clinical application. Second, most of these models do not consider convolutional filters' properties in different feature learning stages. This would generally lead the model to pay attention to a single rough area of the face while ignoring other important areas contributing to depression identification. Therefore, to address these problems, we propose a multi-stage hybrid attention structure that considers the long-range inductive biases in low-level feature learning and high semantic feature representation. Multiple non-overlapping attention regions could be activated simultaneously to capture fined-grain depression features from different facial regions. Experimental results on AVEC 2013 and AVEC 2014 depression datasets illustrate the effectiveness of our method.

## 3. Methodology

### 3.1. Framework overview

The proposed end-to-end depression recognition framework HMHN is presented in Figure 2. To learn high-discriminative attentional features with facial depression details, we first extract the long-range biases between different facial regions by GWA and DFF. Second, the MAB takes the features from the DFF module as input and captures several facial regions with depression information. Then, the AFB module attempts to train these attention maps (i.e., outputs from the MAB module), to focus on non-coincident facial areas and merge these attention maps, which predicts the BDI-II score. In the following, we will describe each component in HMHN detail.

### 3.2. Grid-wise attention

To learn long-range bias in low-level feature extraction of facial images and mine discriminative features with facial depressive patterns without relying on large-scale datasets, motivated by Huang et al. (2021), we introduce the grid-attention mechanism, which mainly includes two parts, local grid feature extraction and grid-wise attention calculation. The details are presented in the following sections.

#### 3.2.1. Local grid feature extraction network

The facial images are cropped and aligned according to their eye positions and resized to 224 × 224 × 3 by the machine learning toolkit Dlib (King, 2009). Then, it divided into *h*×*w* grids before being forwarded to the local grid feature extraction network (LGFE), to extract the depression discrimination information in each grid. The details are as follows:


(1) $$\begin{array}{l} \text{Grid}\left(g,h,w\right)=\left\{g_{1,1}^{C\times \frac{H}{h}\times \frac{W}{w}},...,g_{i,j}^{C\times \frac{H}{h}\times \frac{W}{w}},...\right\} \end{array}$$

(2) $$\begin{array}{l} {\hat{G}}^{hw\times C\times \frac{H}{h}\times \frac{W}{w}}=\text{LGFE}\left(g^{hw\times C\times \frac{H}{h}\times \frac{W}{w}}\right), \end{array}$$

(3) $$\begin{array}{l} {\hat{G}}_{i,j}=\text{LGFE}\left(g_{i,j}\right) \end{array}$$

where *H*, *W*, and *C* are the height, width, and channels of the original image, respectively. $g_{i,j}^{C\times \frac{H}{h}\times \frac{W}{w}}$ represents that the input image *g* is divided into *h*×*w* grids, every grid is with a shape of $C\times \frac{H}{h}\times \frac{W}{w}$ and locates in the *i* th row and the *j* th column in *g*. Next, as shown in the Equations (2) and (3), each grid will be forwarded to the LGFE, and the local depression feature of the facial region learned is defined as ${\hat{G}}_{i,j}$. We believe that every grid features a respective contribution to depression recognition. Therefore, these feature maps are forwarded to the grid-wise attention calculation to weight their importance. The structure of the LGFE is shown in Table 2.

**Table 2:** The configuration of local grid feature extraction network.

**Input**	**Operator**	**Kernel**	**Output**
$C\times \frac{H}{h}\times \frac{W}{w}$	Convolution	1 × 1, Stride 1	$\left(Ck\right)\times \frac{H}{h}\times \frac{W}{w}$
$Ck\times \frac{H}{h}\times \frac{W}{w}$	BatchNorm	/	$\left(Ck\right)\times \frac{H}{h}\times \frac{W}{w}$
$Ck\times \frac{H}{h}\times \frac{W}{w}$	LeakyRelu	/	$\left(Ck\right)\times \frac{H}{h}\times \frac{W}{w}$
$Ck\times \frac{H}{h}\times \frac{W}{w}$	Convolution	1 × 1, Stride 1	$C\times \frac{H}{h}\times \frac{W}{w}$
$C\times \frac{H}{h}\times \frac{W}{w}$	BatchNorm	/	$C\times \frac{H}{h}\times \frac{W}{w}$
$C\times \frac{H}{h}\times \frac{W}{w}$	LeakyRelu	/	$C\times \frac{H}{h}\times \frac{W}{w}$

#### 3.2.2. Grid attention calculation

To better extract the depressive features of facial regions, after the LGFE block, the relationship between different facial regions is constructed through grid attention calculation, which is defined as follows:


(4) $$\begin{array}{l} {\text{Att}}_{q,k}=\delta \left(\frac{q\cdot k}{d_{k}}\right) \end{array}$$

where $d_{k}=\frac{W}{w}$, $q={\hat{G}}^{hw\times C\times \frac{H}{h}\times \frac{W}{w}}$, and $k={\hat{G}}^{hw\times C\times \frac{W}{w}\times \frac{H}{h}}$, and *δ* stand for the softmax operation.

Then, the adaptive average pooling is used to squeeze each channel into a scalar after an attention mechanism and expand the channel back to the original shape. The process is formulated as follows:


(5) $$\begin{array}{l} {\tilde{G}}^{hw\times C\times \frac{H}{h}\times \frac{W}{w}}=Aavp\left({\text{Att}}_{q,k}\right)*Ones\left(\frac{H}{h},\frac{W}{w}\right) \end{array}$$

where “*” represents the scalar matrix product between matrices with a broadcasting property. *Aavp*(·) denoted an adaptive average pooling technique that converts an operand matrix into a scalar and $Ones\left(\frac{H}{h},\frac{W}{w}\right)$ is to generate a matrix with all elements being equal to 1 in the shape of $\left(\frac{H}{h},\frac{W}{w}\right)$.


(6) $$\begin{array}{l} {\tilde{G}}^{C\times H\times W}=Ungrid\left({\tilde{G}}^{hw\times C\times \frac{H}{h}\times \frac{W}{w}}\right)*g^{C\times H\times W} \end{array}$$

where *Ungrid*(·) is the reverse operation of Equation (1), which is used to convert these grid attention maps back to the shape of the original facial image and concat these weights back to the shape of the original matrix.

Thus, the resulting ${\tilde{G}}^{C\times H\times W}$ is a feature map that takes into account the long-range bias between different facial regions in the low-level visual depression feature learning stage.

### 3.3. Deep feature fusion

**Figure 3:**
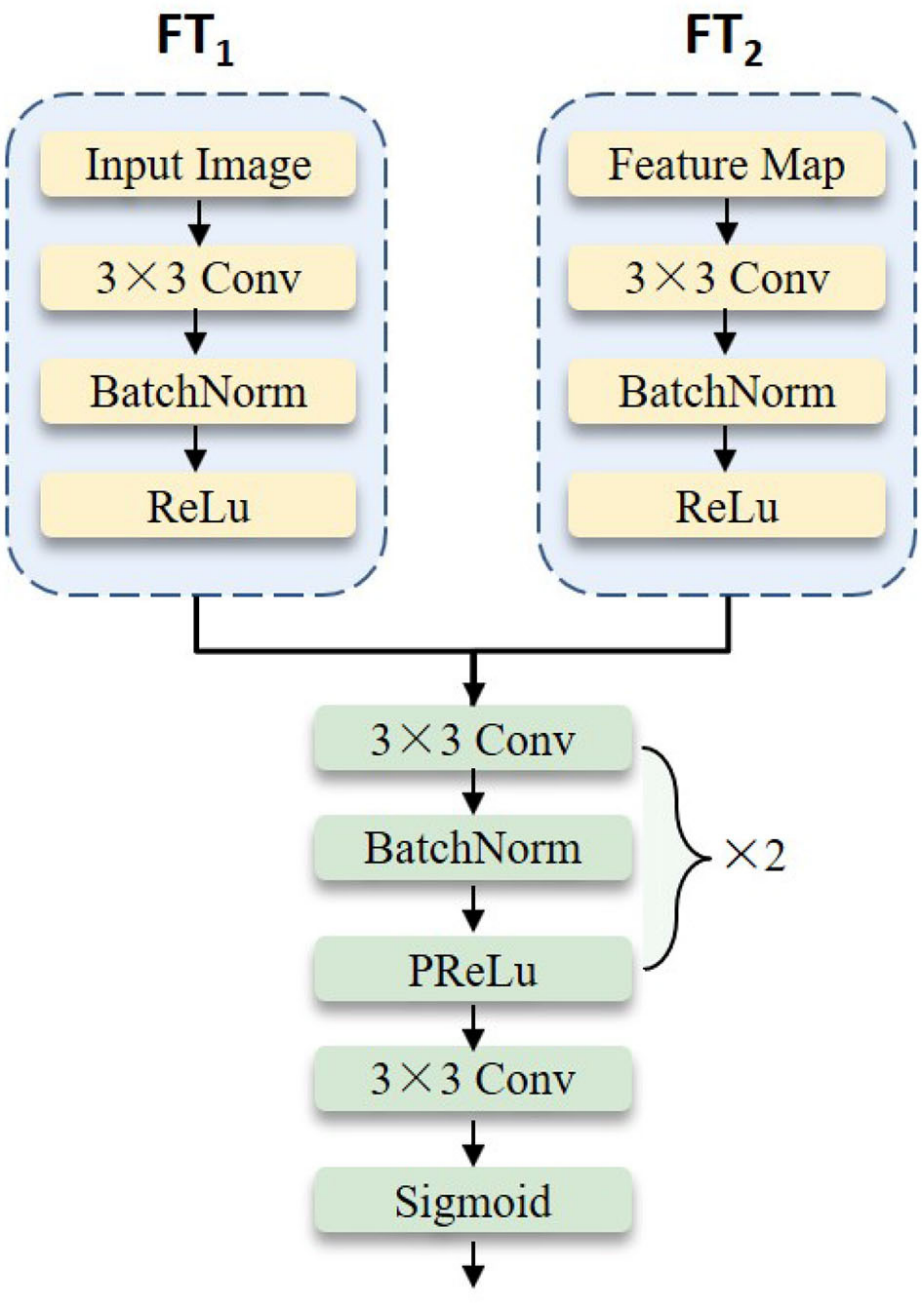
The detailed illustration of the deep feature fusion block.

To further extract the depressive features of the face, we fuse the features between the original image *g* and the weighted feature map $\tilde{G}$ of the backbone network by applying residual network technology. In particular, based on the experimental results in Section 5, we choose to remove the average pooling, flattening, and fully connected layer from ResNet-18 (He et al., 2016) as the backbone. The overall structure of the deep feature fusion block is shown in Figure 3. It mainly includes two feature transformation networks and one feature fusion network. These two feature transformation networks share the structure but not the learning parameters. The mathematical definition is as follows:


(7) $$\begin{array}{l} {\bar{G}}^{C\times H\times W}=\text{DFF}\left({\text{FT}}_{1}\left(g\right)+{\text{FT}}_{2}\left(\tilde{G}\right)\right) \end{array}$$

where **FT**_*i*_(·) (*i*=1,2) is the feature transformation network of the original facial image *g* and the weight feature $\tilde{G}$ extracted from the GWA module, respectively. **DFF** denotes the deep feature fusion network. Finally, the obtained feature map ${\bar{G}}^{C\times H\times W}$ is forwarded to the candidate backbone network.

### 3.4. Multi-head cross attention block

**Figure 4:**
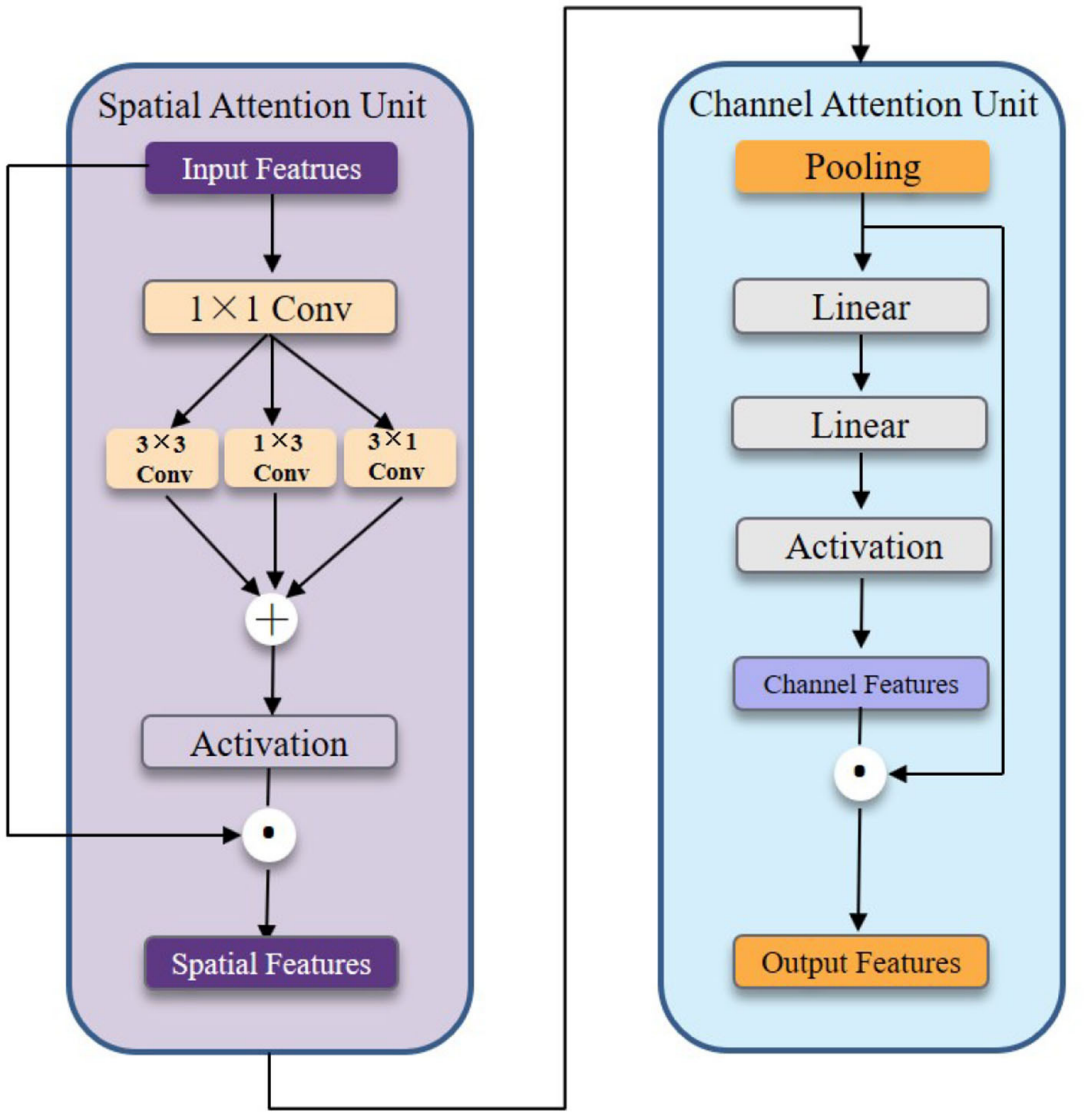
The detailed illustration of the cross attention head.

Facial depression behavior is usually manifested by multiple facial regions simultaneously. The GWA module first extracts the low-level local features of the face in HMHN. Then, we need to encode the high-level interactions between local features by multi-head cross-attention block to achieve a holistic approach. The detailed structure of the MAB block is shown in Figure 4. It is composed of parallel cross-head attention units, which are combinations of spatial and channel attention units that remain independent.

More concretely, The spatial attention unit is shown in the left part of Figure 4. We first feed the input features into the 1 × 1 convolution layer to reduce the channel number. Next, we construct the 3 × 3, 1 × 3, and 3 × 1 convolution kernels to efficiently capture spatial relationships. In general, the spatial attention unit consists of four convolution layers and one activation function to capture local features at multiple scales. The channel attention unit shown in the right part of Figure 4 consists of two linear layers and one activation function. We take advantage of two linear layers to achieve a mini autoencoder to encode channel information.

Mathematically, the above process can be formulated as follows:


(8) $$\begin{array}{l} S_{i}=\bar{G}\times H_{i}\left(\theta_{s},\bar{G}\right),i\in \left\{1,k\right\} \end{array}$$

(9) $$\begin{array}{l} C_{i}=S_{i}\times H_{i}^{\prime }\left(\theta_{c},S_{i}\right),i\in \left\{1,k\right\} \end{array}$$

where *k* is the number of cross attention heads. *H*_*i*_ and $H_{i}^{\prime }$ are defined as the spatial attention head and the channel attention head, respectively, *θ*_*s*_ and *θ*_*c*_ are their parameters. *S*_*i*_ and *C*_*i*_ represent the output of the *i*-h spatial attention and channel attention, separately.

### 3.5. Attention fusion block

After going through several modules above, our HMHN has been able to capture subtle facial depression features, but the multi-head construction could not learn attention maps in an orchestrated fashion. In other words, we hope that different branches can focus on different facial regions as much as possible and fuse the depression feature information of each head. To achieve this aim, we propose that the AFB enhance further the features learned by MAB. In the meantime, the cross-attention heads are supervised to center on different critical regions and avoid overlapping attention using the partition loss, which is defined as follows:


(10) $$\begin{array}{l} L_{sum}=L_{att}+L_{mse} \end{array}$$

(11) $$\begin{array}{l} L_{att}=\frac{1}{NC}\overset{N}{\underset{i=1}{\sum }}\overset{C}{\underset{j=1}{\sum }}\log\left(1+\frac{k}{\sigma_{ij}^{2}}\right) \end{array}$$

This loss contains two components, where $L_{mse}$ is the square loss for regression and $L_{att}$ is partition loss to maximize the variance among the attention maps, *k* is the number of cross attention, *N* is the number of samples, *C* is the channel size of the attention maps, and $\sigma_{ij}^{2}$ is denoted the variance of the *j*-th channel on the *i*-th sample. The merged attention map is then used for computing the BDI-II score with a regression output layer. Finally, we learn the deep discriminative features by jointly minimizing the unified loss functions $L_{sum}$.

## 4. Experiments

In order to demonstrate the effectiveness of our depression recognition approach, we conducted experiments on two publicly available datasets, namely AVEC 2013 and AVEC 2014. Compare our performance with start-of-the-art methods, and demonstrate the effectiveness of each component in our model by an ablation study. This section presents a description of the dataset, data pre-processing, experimental setting and evaluation metrics.

### 4.1. AVEC 2013 and AVEC 2014 datasets

**Figure 5:**
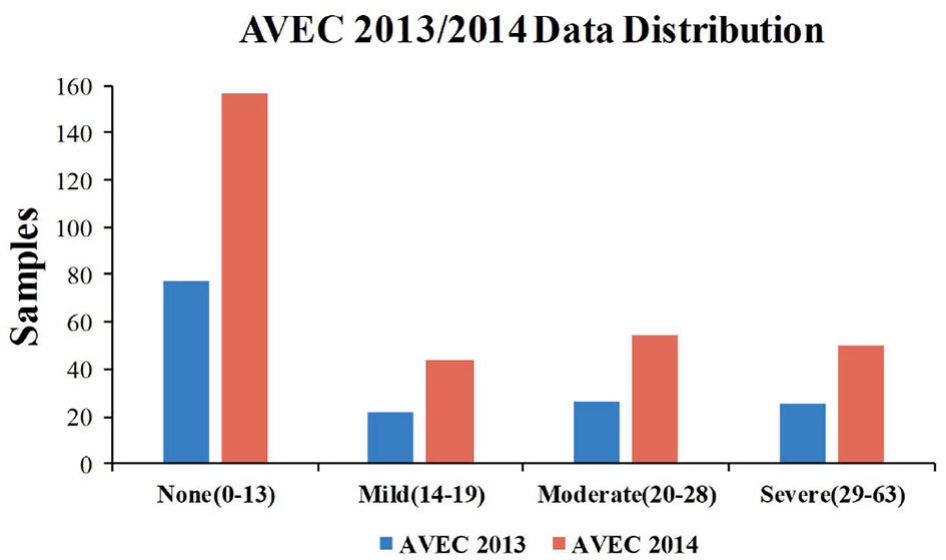
The distribution of BDI-II scores in the AVEC 2013 and AVEC 2014 datasets.

In the present paper, all experiments are evaluated on AVEC 2013 and AVEC 2014 depression datasets. The distribution of the BDI-II scores in both the AVEC 2013 and AVEC 2014 datasets is shown in Figure 5.

For the AVEC 2013 depression dataset, there are 150 video clips recorded by 82 subjects participating in human-computer interaction (HCI) task with a microphone and a webcam to record the information. The age range for all subjects in the dataset is 18–63 years old, with an average age is 31.5 years old and a standard deviation of 12.3 years. These video recordings are set to 30 frames per second (fps) with a resolution of 640 × 480 pixels. This depression dataset has been divided into three partitions by the publisher, i.e., training, development, and test set. For every partition, it has 50 videos, and each video has a label corresponding to its depression severity level, which is assessed based on the BDI-II questionnaire.

The AVEC 2014 depression dataset is a subset of the AVEC 2013 dataset. There are two tasks included: FreeForm and Northwind, both of which have 150 video clips. Specifically, in the “FreeForm” task, the subjects responded to several questions, such as describing a sad childhood memory or saying their favorite dish. In the “Northwind” task, the subjects are required to read an excerpt audibly from a fable. The same as AVEC 2013, it also has three partitions, i.e., training, development, and test sets. We perform experiments employing training and development sets from both tasks as training data, and the test sets are used to measure the performance of the model.

### 4.2. Experimental settings and evaluation metrics

#### 4.2.1. Experimental settings

The overall framework of HMHN is shown in Figure 2. A machine learning toolkit DliB (King, 2009) is adopted to resize the generated facial images to 224 × 224 with RGB color channels. Instead of using a pre-trained architecture to predict depression severity, we directly train the whole framework in an end-to-end fashion. To be specific, our experimental code is implemented with Pytorch (Paszke et al., 2019), and the models are trained on a local GPU server with a TESLA-A100 GPU (40 G global memory). In order to obtain fast convergence, we use the AdamW (Loshchilov and Hutter, 2017) optimizer with an adaptive learning rate strategy, and its initial learning rate is 0.001, The batch size is 64, the dropout rate is 0.2, and the learning factor is set to 0.1.

#### 4.2.2. Evaluation metrics

The performance of the baseline models is assessed on AVEC 2013 and AVEC 2014 datasets in terms of two evaluation metrics—Mean Absolute Error (MAE) and Root Mean Square Error (RMSE). Afterward, many researchers have been adopting these two metrics to evaluate the prediction accuracy of their works. This study also regards RMSE and MAE as the metrics during testing to make an equitable comparison, which details are defined as:


(12) $$MAE=\frac{1}{M}\sum_{j=1}^{M}\left|{\hat{\ell }}_{j}-\ell_{j}\right|$$

(13) $$\begin{array}{l} RMSE=\sqrt{\frac{1}{M}\overset{M}{\underset{j=1}{\sum }}{\left({\hat{\ell }}_{j}-\ell_{j}\right)}^{2}} \end{array}$$

where *M* is the total number of video samples, ℓ_*j*_ and ${\hat{\ell }}_{j}$ are the ground truth and the predicted BDI-II score of the *j*-th subject, respectively.

## 5. Experimental results and discussion

In this section, we first perform an ablation study to examine the effectiveness of individual components in the propose framework. Then, we compare the architecture with several state-of-the-art vision-based depression analysis methods to show its promising performance.

### 5.1. Ablation study

In order to verify the effectiveness of the proposed HMHN, we carry out the ablation studies on AVEC 2013 and AVEC 2014 datasets to assess the efficacy of critical components in our method. The results are shown in Tables 3, 4. Specifically, Resnet18+GWA (*B*1,*B*2) outperforms the backbone network (*A*1,*A*2) on both datasets owing to GWA can learn long-range bias in low-level features of facial images. *D*1 and *D*2 are improved by MAB and AFB, which capture multiple non-overlapping attention simultaneously. *E*1 and *E*2 integrate all modules, yielding better results than using them separately. This observation demonstrates that the multi-stage attention mechanism performs better than the one-stage attention mechanism. The prediction accuracy of depression level can be effectively improved by encoding the low-level to high-level interactions between depression discriminative features of multiple facial regions.

**Table 3:** Ablation study of the individual components on the test set of AVEC 2013.

**Combination**	**Evaluation metrics**	
	**MAE**	**RMSE**
**A1**: Resnet18 (backbone)	8.47	9.32
**B1**: Resnet18+GWA	7.68	8.31
**C1**: Resnet18+GWA+DFF	7.49	8.29
**D1**: Resnet18+MAB+AFB	6.88	7.91
**E1**: Resnet18+DFF+GWA+MAB+AFB (Ours)	**6.05**	**7.38**

**Table 4:** Ablation study of the individual components on the test set of AVEC 2014.

**Combination**	**Evaluation metrics**	
	**MAE**	**RMSE**
**A2**: Resnet18 (backbone)	8.38	9.13
**B2**: Resnet18+GWA	7.57	8.47
**C2**: Resnet18+GWA+DFF	7.41	8.46
**D2**: Resnet18+MAB+AFB	6.90	8.13
**E2**: Resnet18+DFF+GWA+MAB+AFB (Ours)	**6.01**	**7.60**

The bold values indicate the best results.

### 5.2. Number of the cross attention heads

**Figure 6:**
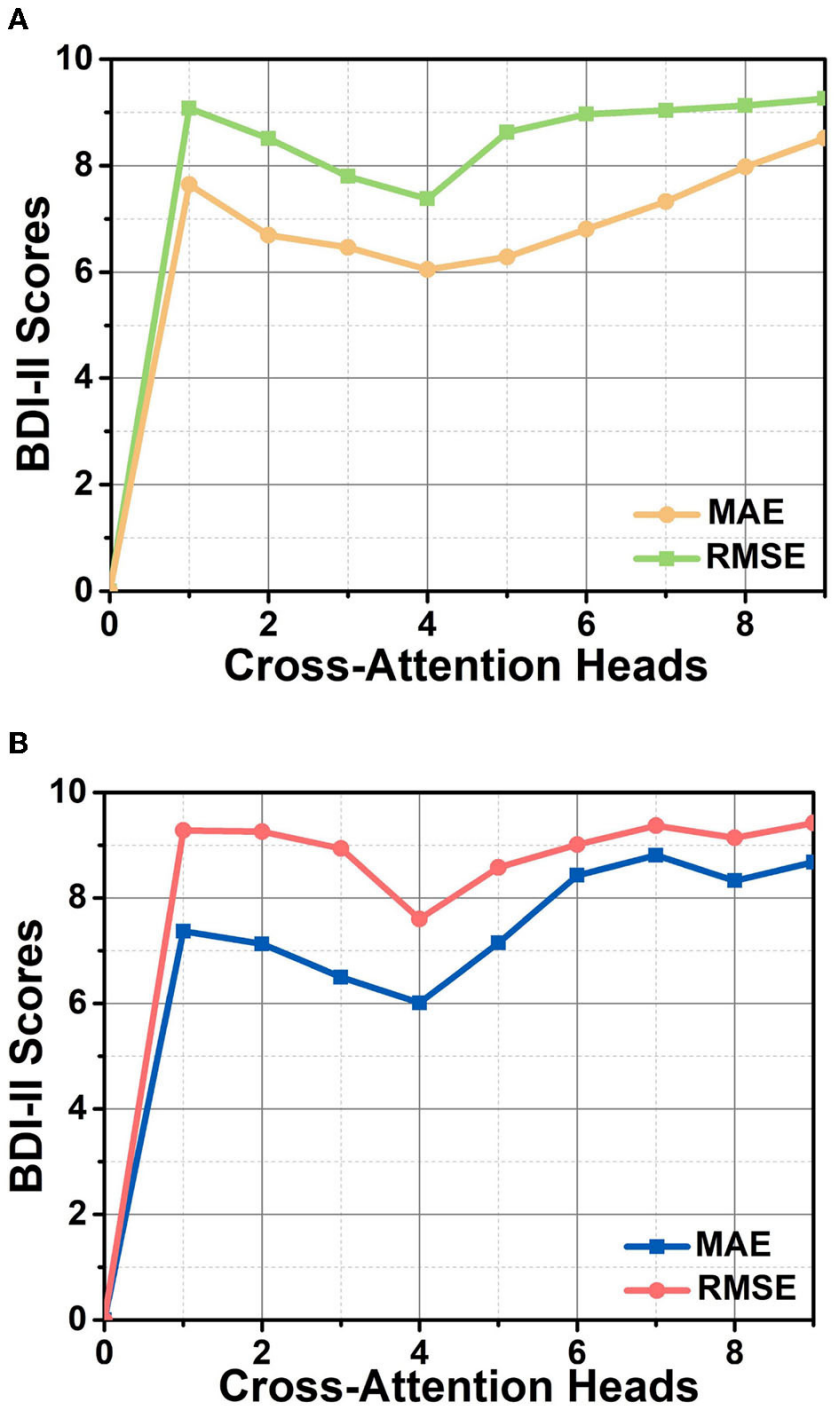
The performance of the HMHN architecture in terms of RMSE and MAE for various sizes of cross-attention head on AVEC 2013 **(A)** and AVEC 2014 **(B)** datasets.

We opt different numbers of cross-attention heads to observe their effect on the depression recognition performance of the model, allowing us to select an optimal cross-attention head size. The results are shown in Figure 6, where the lines with different colors represent the two evaluation metrics, RMSE and MAE, respectively. The top and bottom figures indicate experimental results on two different datasets, AVEC 2013 and AVEC 2014. It is apparent that the increasing number of layers does not imply an improvement in the performance, and equipping four cross-attention heads maximizes the model's performance. It is probably related that facial depression recognition is affected by multiple facial regions. The single attention head cannot sufficiently capture all the subtle and complex appearance variations, while too many attention heads make the attention regions overly distributed. As shown in Figure 6, our method explicitly learns to attend to multiple local image regions for facial depression recognition.

### 5.3. Impact of the grid size

**Figure 7:**
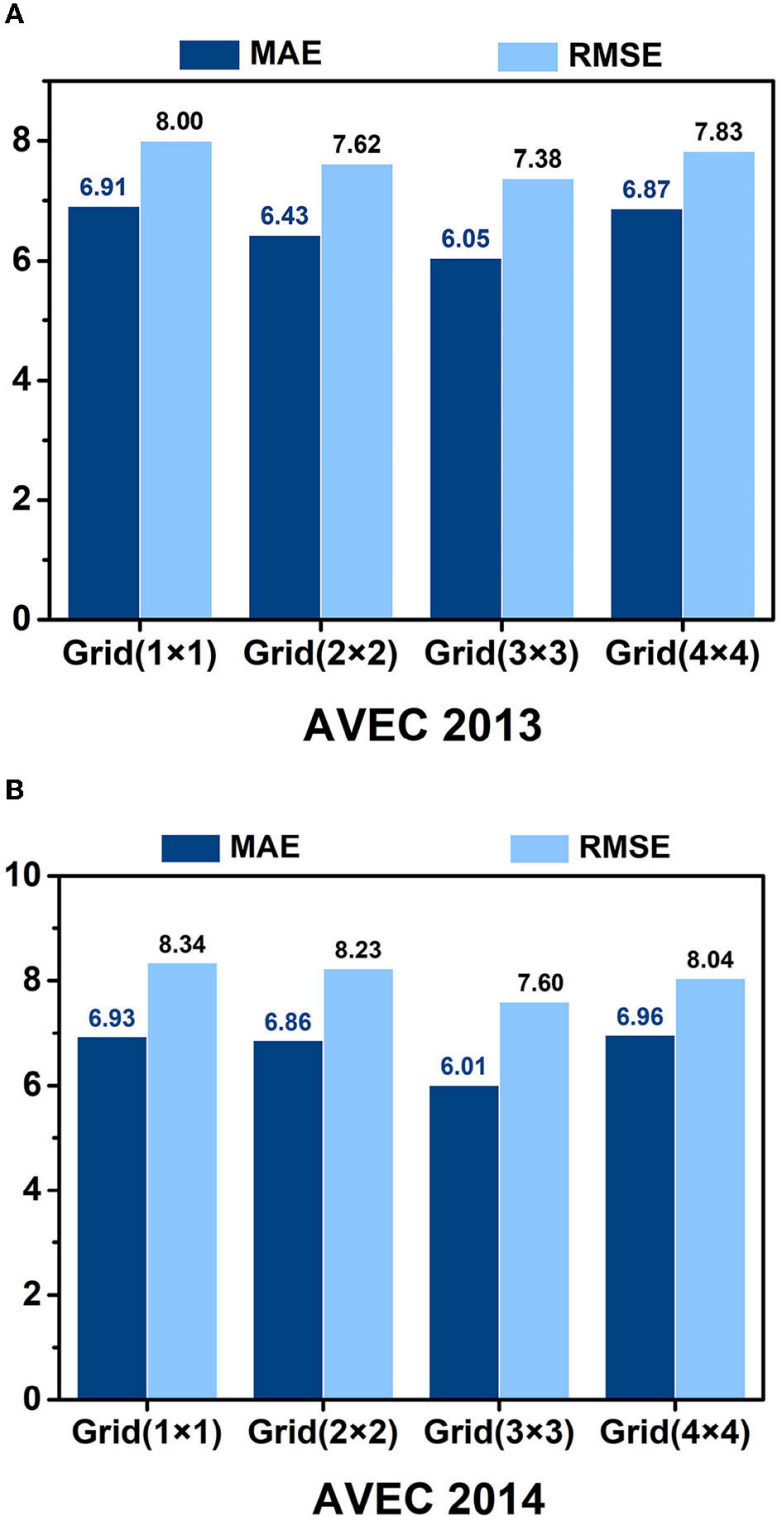
Recognition results of different grid parameters of the HMHN model on the AVEC 2013 **(A)** and AVEC 2014 **(B)** datasets.

We examine the impact of grid parameters on the model's performance, as evidenced in Figure 7. Our findings indicate that utilizing a grid strategy generally leads to improved performance over not using a grid strategy. The **Grid**(3 × 3) achieves the best results among the tested grid parameters, with an MAE of 6.05 and an RMSE of 7.38 on the AVEC 2013 dataset, and MAE = 6.01 and RMSE = 7.60 on the AVEC 2014 dataset. This phenomenon may be related to the spatial position and size of the grid, as an overly large or small grid size may limit the expression ability of the receptive field and interfere with the acquisition of depression information across facial regions.

### 5.4. Kernel size of separable convolutions

We conduct experiments to evaluate the effect of separable convolutions in MAB modules. We test standard convolutions and separable convolutions with different kernel sizes. According to our experimental results, as shown in Table 5, using a separable convolution model with a smaller kernel size (1 × 3, 3 × 3, 3 × 1) performs better than using a larger kernel size such as (1 × 7, 7 × 7, 7 × 1) and (1 × 5, 5 × 5, 5 × 1). In addition, we also find that separable convolutions can achieve similar performance with fewer parameters than standard convolutions. For example, on the AVEC 2013 dataset, the MAE of the separable convolution model with convolution kernel sizes (1 × 3, 3 × 3, 3 × 1) is 6.05, and the RMSE is 7.38. Compared with using standard convolution, the number of separable convolution parameters is reduced by 32.8%.

**Table 5:** Kernel size of separable convolution on AVEC 2013 and AVEC 2014 datasets.

**Kernel settings**	**Params(M)**	**AVEC 2013**		**AVEC 2014**	
		**MAE**	**RMSE**	**MAE**	**RMSE**
Standard Conv	29.33	6.07	7.43	6.09	7.66
(1 × 7, 7 × 7, 7 × 1)	26.57	6.16	7.49	6.19	7.78
(1 × 5, 5 × 5, 5 × 1)	22.63	6.14	7.51	6.12	7.71
(3 × 1, 1 × 3)	17.78	6.21	7.56	6.27	7.83
(3 × 3, 1 × 3, 3 × 1)	19.72	**6.05**	**7.38**	**6.01**	**7.60**

The bold values indicate the best results.

### 5.5. Comparison with state-of-the-art methods

In order to further demonstrate the depressive recognition performance of the proposed model, We present the quantitative performance comparison results in Tables 6, 7 for AVEC 2013 and AVEC 2014, respectively. Specifically, models in Valstar et al. (2013, 2014), Wen et al. (2015), He et al. (2018), and Niu et al. (2019) are based on hand-crafted representations. Our method outperforms all other methods, mainly because hand-crafted features rely on researchers' experiences, and it is difficult to characterize depression cues fully. At the same time, our HMHN uses deep neural networks and the multi-attention stage mechanism, which can capture complete semantic information, thereby improving the prediction performance.

**Table 6:** Depression level prediction performance compared with different methods on the AVEC 2013 test set.

**Category**	**Methods**	**MAE**	**RMSE**
Pre-trained	Valstar et al. (2013)/LPQ	10.88	13.61
	Cummins et al. (2013)/PHOG	/	10.45
	Wen et al. (2015)/LPQ-TOP	8.22	10.27
	He et al. (2018)/MRLBP-TOP, DPFV	7.55	9.20
	Niu et al. (2019)/LSOGCP	6.97	9.17
	Zhu et al. (2017)/Optical Flow, 2D-CNN	7.58	9.82
	Al Jazaery and Guo (2018)/C3D, RNN	7.37	9.28
	De Melo et al. (2019)/ResNet-50	6.30	8.25
	Zhou et al. (2020)/2D-CNN	6.20	8.28
	De Melo et al. (2020)/Two-Stream	**5.96**	7.97
	Uddin et al. (2020)/LSTM	7.04	8.93
	De Melo et al. (2021)/MDN	6.59	8.39
	Niu et al. (2022)/2D-CNN	6.12	7.49
	He et al. (2022a)/2D-CNN	7.36	9.17
End-to-end	He et al. (2021a)/2D-CNN, Attention	6.59	8.39
	He et al. (2021b)/3D-CNN	6.83	8.46
	He et al. (2022b)/2D-CNN	7.02	9.37
	Liu et al. (2023)/2D-CNN, Attention	6.08	7.59
	Ours	**6.05**	**7.38**

The bold values indicate the best results.

**Table 7:** Depression level prediction performance compared with different methods on the AVEC 2014 test set.

**Category**	**Methods**	**MAE**	**RMSE**
Pre-trained	Valstar et al. (2014)/LGBP-TOP	8.86	10.86
	Dhall and Goecke (2015)/LBP-TOP	7.08	8.91
	He et al. (2018)/MRLBP-TOP, DPFV	7.21	9.01
	Niu et al. (2019)/LSOGCP	7.19	9.10
	Zhu et al. (2017)/Optical Flow, 2D-CNN	7.47	9.55
	Al Jazaery and Guo (2018)/C3D, RNN	7.22	9.20
	De Melo et al. (2019)/ResNet-50	6.13	8.23
	Zhou et al. (2020)/2D-CNN	6.21	8.39
	De Melo et al. (2020)/Two-Stream	6.20	7.94
	Uddin et al. (2020)/LSTM	6.86	8.78
	De Melo et al. (2021)/MDN	6.06	7.65
	Niu et al. (2022)/2D-CNN	**6.01**	**7.56**
	He et al. (2022a)/2D-CNN	7.26	9.03
End-to-end	He et al. (2021a)/2D-CNN, Attention	6.51	8.30
	He et al. (2021b)/3D-CNN	6.78	8.42
	He et al. (2022b)/2D-CNN	6.95	9.24
	Liu et al. (2023)/2D-CNN, Attention	6.04	7.98
	Ours	**6.01**	**7.60**

The bold values indicate the best results.

For the methods using deep neural networks, Zhu et al. (2017), Al Jazaery and Guo (2018), Zhou et al. (2020), and He et al. (2022a) train the deep models on a large dataset and then fine-tune on the AVEC 2013 and AVEC 2014 datasets. HMHN is an end-to-end scheme for depression recognition and achieves an impressive performance even without a pre-trained model on other large-scale datasets. As shown in Tables 6, 7, we achieve the best performance among end-to-end methods on the AVEC 2013 (MAE = 6.05, RMSE = 7.38) and AVEC 2014 (MAE = 6.01, RMSE = 7.60) datasets. We also achieve the second-best performance compared to other methods pre-trained on large-scale datasets. Specifically, Zhou et al. (2020) propose a CNN-based visual depression recognition model by roughly dividing the facial region into three parts and then combined with the overall facial image to improve the recognition performance of the model. Our better performance is due to the multi-stage attention mechanism for the extraction of depressive features, and Zhou et al.'s visualization results show that their model focuses attention on only one region and ignores other facial details that contribute to depression recognition. In contrast, He et al. (2021a) achieves a passable performance without a pre-trained model. The authors divide the facial region by facial landmark points, then block the feature map to extract local feature information. Finally, the feature aggregation method is used to automatically learn the facial region's local and global feature information. He et al. (2021b, 2022b) and Liu et al. (2023) are also end-to-end methods. Our HMHN outperforms those methods by a significant margin. One important reason is that we consider the long-range inductive biases in both low-level feature learning and high-semantic feature representation. At the same time, Niu et al. (2022) improve the prediction accuracy of depression levels by investigating the correlation between channels and vectorizing the tensors along the time and channel dimensions. De Melo et al. (2020) to encode the smooth and sudden facial expression variations to assess individual BDI-II scores. These two methods model the spatio-temporal information of facial regions; our propose is trained from scratch using only facial visual information and achieves comparable results.

### 5.6. Visual analysis

In order to intuitively observe how the model predicts depression scores from facial images, we present the visualized facial images with different cross-attention heads in Figure 1. The first column of Figure 1 shows the original images, and the second to fifth columns represent the attention regions of different cross-attention heads. The heatmap in the faces is the focus area learned by the model. Our model can attend to multiple locations simultaneously before fusing the attention maps. Our HMHN model specifically focuses on the facial muscle movement regions related to depression, such as the mouth, eyebrows, and eyes, while suppressing irrelevant regions.

## 6. Conclusion

In this paper, an end-to-end two-stage attention mechanism architecture named HMHN for predicting an individual's depression level by facial images is proposed. HMHN can focus on multiple depression feature-rich areas of the face yet is remarkably capable of recent works in recognition. Specifically, this model mainly includes four blocks: the grid-wise attention block (GWA), deep feature fusion block (DFF), multi-head cross attention block (MAB), and attention fusion block (AFB). GWA and DFF are the first stages to capture the dependencies among different regions from a facial image in a way that the parameter learning of convolutional filters is regularized. In the second stage, the MAB and AFB block is composed of parallel cross-head attention units, which combine spatial and channel attention units to obtain final facial depression features bbsy encoding higher-order interactions between local features. Experimental results on AVEC 2013 and AVEC 2014 depression datasets show the effectiveness of video-based depression recognition of the proposed framework when compared with most of the state-of-the- art approaches.

In the future, we will collect and build a dataset with more depression patients to learn more robust feature representations from the images of diverse appearances. In addition, investigation of the multi-modal (audio, video, text, etc.) depression representation learning appears to be an attractive topic.

## Data availability statement

The original contributions presented in the study are included in the article/supplementary material, further inquiries can be directed to the corresponding authors.

## Author contributions

YL and ZL conceived the study design. YL analyzed the experimental data and drafted the manuscript. ZS, LZ, and XY helped to interpret the data analysis. XH and BH were responsible for the overall planning of the dissertation. All authors agree to be accountable for the content of the work. All authors contributed to the article and approved the submitted version.
